# RedLionfish – fast Richardson-Lucy Deconvolution package for efficient point spread function suppression in volumetric data

**DOI:** 10.12688/wellcomeopenres.21505.1

**Published:** 2024-06-03

**Authors:** Luís M. A. Perdigão, Casper Berger, Neville B.-Y. Yee, Michele C. Darrow, Mark Basham

**Affiliations:** 1 The Rosalind Franklin Institute, Didcot, OX11 0DE, UK

**Keywords:** Python, Image processing, Microscopy, napari, Fluorescence Light Microscopy, Correlative imaging

## Abstract

The experimental limitations with optics observed in many microscopy and astronomy instruments result in detrimental effects for the imaging of objects. This can be generally described mathematically as a convolution of the real object image with the point spread function that characterizes the optical system. The popular Richardson-Lucy (RL) deconvolution algorithm is widely used for the inverse process of restoring the data without these optical aberrations, often a critical step in data processing of experimental data. Here we present the versatile RedLionfish python package, that was written to make the RL deconvolution of volumetric (3D) data easier to run, very fast (by exploiting GPU computing capabilities) and with automatic handling of hardware limitations for large datasets. It can be used programmatically in Python/numpy using conda or PyPi package managers, or with a graphical user interface as a napari plugin.

## Plain language summary

In order to observe biological phenomena at microscopic scale, light or fluorescent microscopes are often used. These instruments use optical devices such as lenses and mirrors to guide light and help form an image that can be recorded and analyzed. Modern optical methods and techniques have been developed so that scientists can obtain 3D images of microscopic objects of interest, such as confocal microscopy or light sheet microscopy. Currently, optical instruments can readily observe cells and their contents down to a few nanometers resolution (e.g.: chromosomes). However, there are physical limitations that prevent the resolution of images below a nanometer. One such limitation comes from the inherent property of light itself as an electromagnetic wave with wavelengths in the hundreds of nanometers range. Another major limitation comes from the guiding optics used to both illuminate the object and to probe the samples being studied. This results in images that are unavoidably blurry preventing differentiation of small, nearby details. Fortunately, with a good understanding of what causes the blurriness, it is possible to use a filter to reverse it, and recover the image to closely match its non-blurred form. This filter is widely used by scientists and is called the Richardson-Lucy deconvolution algorithm. Although this filter is widely available in many scientific software packages, its implementation is often slow and limited to particular imaging analysis applications, with poor programmatic access. With the popularity of the Python programming language, and an open-source image viewer (napari) we have developed the Redlionfish package to apply the RL filter to 3D image data in a speed optimized manner, while also being easy to use and to install.

## Introduction

A pseudo-mathematical description of the image formation and restoration begins by considering a scenario where a user wants to acquire a perfectly sharp picture of an object with a camera/detector. Even when the object is in focus, there are additional instrumental effects and physical limitations that blur the data and add noise to the measured image. Mathematically, this blurring process can be described as being the result of the original image convoluted with the point spread function (PSF) characteristic of the measuring instrument. The noise is often assumed to be a Poisson noise (also known as shot noise) ^1^, but its nature will not be elaborated here.

In three-dimensions, such as the data collected by a confocal microscope in a z-stack experiment, or in light-sheet microscopy experiment, the effect of the PSF-blurring and added noise in the measured image can be modelled by:


$$\begin{array}{l} MI(x,y,z)=\int_{V}OI(x_{0},y_{0},z_{0})\cdot PSF(x-x_{0},y-y_{0},z-z_{0})dx_{0}dy_{0}dz_{0}+N(x,y,z)\\ \;=OI*PSF+N(x,y,z) \end{array}\;(Eq. 1)$$

with *MI* being the Measured Image, *OI* being the Object Image, *N*( *x*, *y*, *z*) being experimental noise, and ( *x*, *y*, *z*) the continuous coordinates in three dimensions. In discrete form (pixels or voxels with coordinates ( *i*, *j*, *k*) this can be approximated as:


$$\;MI_{i,j,k}=\sum_{i_{0}}\sum_{j_{0}}\sum_{k_{0}}OI_{i_{0},j_{0},k_{0}}\cdot PSF_{i,i_{0},j,j_{0},k,k_{0}}+N_{i,i,k}\;(Eq. 2)$$

where *PSF*_*i*, *i*_0_, *j*, *j*_0_, *k*, *k*_0__ is usually a function of *i* – *i*_0_, *j* – *j*_0_, and *k* – *k*_0_. In the approximation where *N*_*i*, *i*, *k*_ ≈ 0 the measured image is simply the mathematically discrete convolution of *OI* and *PSF*.

What is often desired is to retrieve the original image from measured data, so the question is how to reverse the process (deconvolve) and extract the *OI*_*i*_0_, *j*_0_, *k*_0__, when starting with an experimentally acquired *MI*_*i*, *j*, *k*_ and known PSF. It is tempting to deconvolve by using the Fourier transform's convolution theorem, namely using the formula:


$$\;OI=FFT^{-1}\left(\frac{FFT(MI)}{FFT(PSF)}\right)\;(Eq. 3)$$

If there was no noise this would be mathematically correct, but even if we assume that it is negligible the result obtained is often of poor quality because this added noise is greatly amplified through the division in this formula. A better solution is to use the Richardson-Lucy (RL) iterative algorithm for deconvolution. The Richardson-Lucy iterative algorithm was developed independently by Richardson ^2^, by Lucy ^3^ and, equivalently, by others using maximum-likelihood estimation ^4, 5^ and is a well proven and documented computational method for improving experimental images or data, and widely used in microscopy and astronomy ^6^. The algorithm can suppress both the PSF and noise in order to obtain a good approximation for the object being imaged. Mathematical deduction of the formula takes a probabilistic interpretation of the image data, hence the Bayes theorem can be used to calculate the inverse operation in probabilistic terms. From Richardson’s article ^2^, and renaming variables here, the one-dimensional form of the restorative iteration in its discrete form is:


$$\;EI_{n+1,i}=EI_{n,i}\times \sum_{i_{0}}PSF_{i,i_{0}}\frac{MI_{i_{0}}}{\sum_{i_{1}}PSF_{i_{1},i_{0}}\cdot EI_{n,i_{1}}}\;(Eq. 4)$$

With *EI*_*n*+1, *i*_ and *EI _*n*, *i*_* being the estimated image at iterations *n* + 1 and *n* respectively.

This formula can be written as convolutions, but it is important to be careful with the PSF indices meaning that in one of the convolutions the PSF data must be *flipped*.


$$\;EI_{n+1}=EI_{n}\times \left[FSP*\frac{MI}{PSF*EI_{n}}\right]\;(Eq. 5)$$

with *FSP* being the *flipped* form of the *PSF* discrete data and * representing the convolution operation. Clearly, each iteration and consequently the whole algorithm, requires a known PSF. This formula is also valid in higher dimensions, with the convolution, PSF flipping and element-wise multiplication being the multi-dimensional equivalents. We focus our discussion on the three-dimensional case and how to implement and optimize this calculation.

Experimental three-dimensional data is becoming increasingly common, with tomography ^7^ and light-sheet microscopy ^8^ being two notable examples. RL-deconvolution is often an integral part of the data analysis workflow, but it is quite time-consuming and resource hungry, in particular when dealing with large data sets. As such, fast and reliable RL-deconvolution processing can be useful in accelerating data processing. We first note that each iteration involves the calculation of two convolutions, one multiplication and one division (see Equation 2). Computationally, the convolution is known to be the slowest since each voxel in the original data is multiplied with each voxel in the PSF, applied to all voxels. The convolution calculation is commonly accelerated using the fast Fourier transform (FFT). A single convolution calculation involves three FFTs (two forward and one inverse) and a multiplication calculation. This is significantly faster than calculating the convolution by using the conventional summation convolution formula (direct convolution) ^9^. Despite this algorithmic shortcut, running 10 iterations on a modestly sized volume with 1024×1024×64 pixels, and a PSF of 64×64×64 pixels can take up to 10 mins. It may also introduce unwanted circularity in the convolution due to the fact that FFTs assume the data is periodic, as discussed later.

An additional problem is that the intermediate calculations such as the FFT and other mathematical operations require intermediate storage of the arrays in memory as floating point numbers. With restricted GPU or CPU memory this iterative calculation is likely to throw out-of-memory errors when handling large data volumes. Access to supercomputers may not be the most convenient solution sought for a preprocessing filter, as it is usually desirable to view the result of the process as soon as possible after the data collection.

## Methods

### Implementation and architecture

RedLionfish package was created to address most of the limitations described above. It is optimized to be fast, by exploiting the availability of general-purpose computing in graphic processing units (GPGPU) hardware. It uses PyOpenCL ^10^ through another package called Reikna ^11^ which conveniently includes FFT kernels. Since it runs in OpenCL it is also cross-compatible with most CPUs and GPUs, and not restricted to NVIDIA cards, unlike libraries which use CUDA for their implementations. It is important to note here that OpenCL can run in both GPU and in CPU, depending on the choice. Redlionfish by default uses the first platform available through an internal query, but this can programmatically be adjusted to choose a preferential platform by matching a user provided string such as “intel” or “nvidia”. As a failsafe, RedLionfish also has a CPU version of the RL iterative algorithm, optimized for speed. To address potential out-of-memory issues in the GPU calculations, the RL deconvolution can run in blocks (or chunks), removing size limitations of the calculation. To facilitate access to this utility, this package has been made available in PyPi, in anaconda environments with condaforge, and as a napari plugin ^12, 13^.

**Figure 1.**
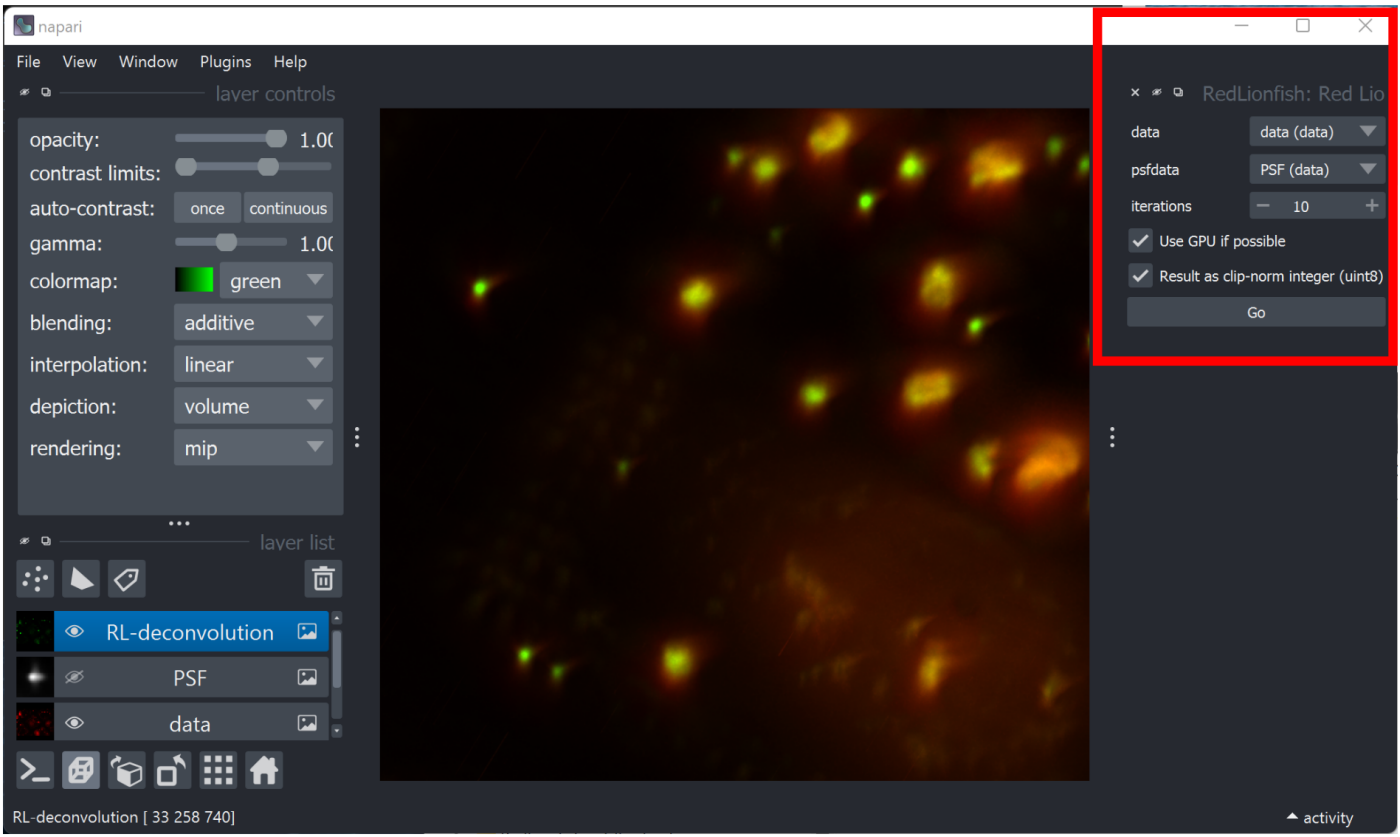
Screenshot of RedLionfish user interface in Napari software with the widget controls marked with a red box.

To address different ways that users may wish to use this deconvolution there are two major options:

- **napari plugin**: RedLionfish package was made into a simple napari plugin using the MagicGUI. Required inputs are the image data, PSF, number of iterations and optional GPU usage ( Figure 1). It can be installed through the napari plugins engine.
- **Programmatically**. Coding in Python makes RedLionfish easy to use programmatically as shown in the example files provided with the package, including Jupyter Notebooks ^14^ which are excellent for prototyping. Upon package installation in the current Python environment, the package is accessible using the *import RedLionfishDeconv* command. The simplest way to run a RL deconvolution on a numpy 3D data array with a given PSF, is by running the function *doRLDeconvolutionFromNpArrays()* with the appropriate parameters. Other specific CPU, GPU or block deconvolution functions are also available. If a user requires debugging information in the calculation progress this can enabled by setting *logging.basicConfig(level=logging.INFO)*. The ability to run programmatically, and the permissive Apache V2.0 license, also means that RedLionfish can be included in other packages, such as the correlative image processing software called 3DCT ^15^ (see https://github.com/rosalindfranklininstitute/3DCT).

An additional functionality that is included is the ability to monitor calculation progress through a callback method. The full RL-deconvolution calculation can take a while to complete and once launched the interactive software may feel like it is hanging. An optional callback method can be passed to the calculation that is called once every iteration has completed or, in case of block deconvolution, in every completion of a block. This is useful to reassure the user that the calculation is still running.

**Figure 2.**
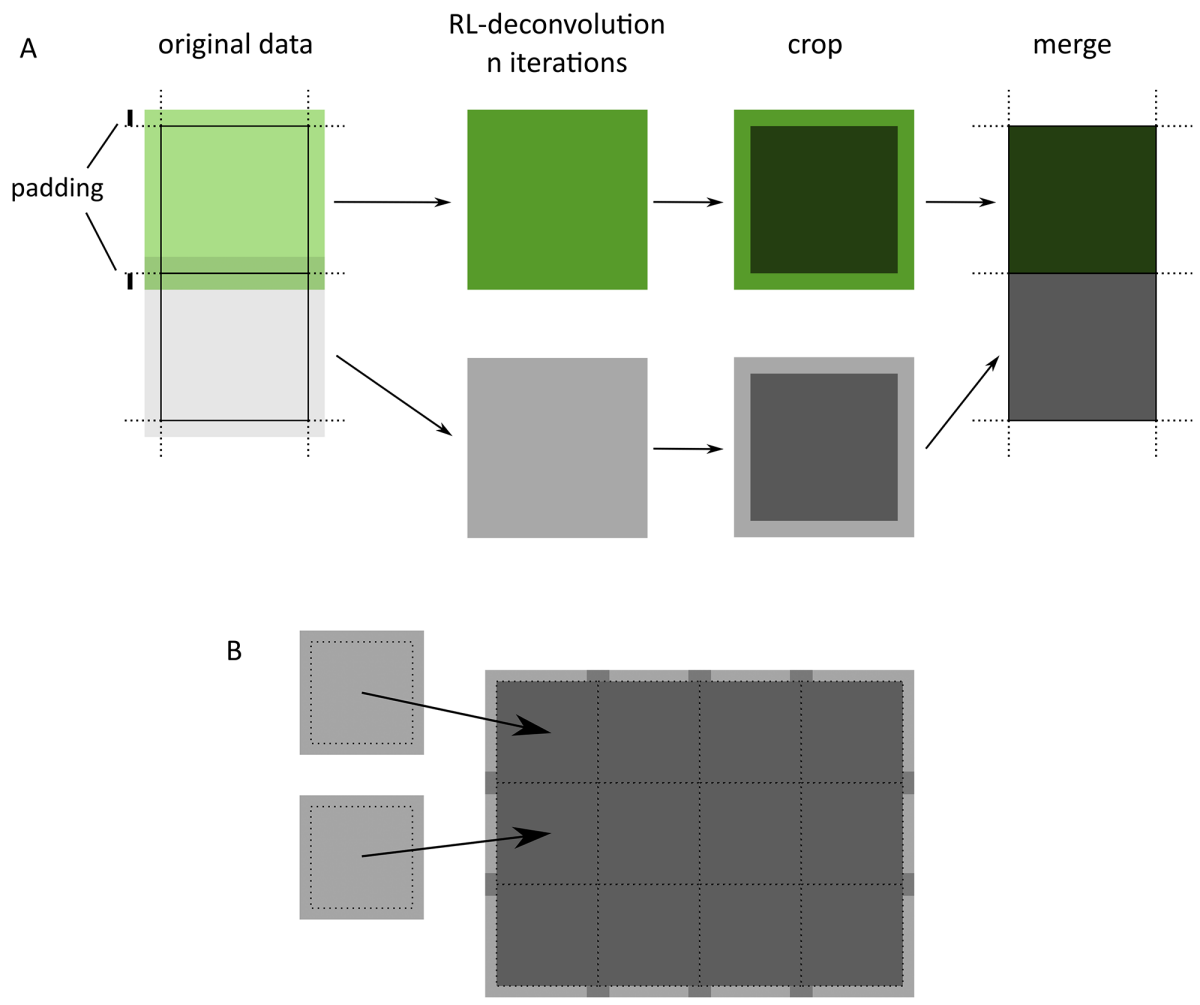
Simplified schematic of the block algorithm for the OpenCL implementation of the RL deconvolution. ( **A**) Data is split into extended blocks. The complete iterative calculation is performed in each block. Then the result is cropped and merged to a single data volume. ( **B**) Illustration of merging multiple calculation blocks onto the full result, showing that only central region (most valid) is used. Note that edges of the volume are treated as if the volume was not split, so these edges are not padded or cropped.

Using a fast implementation of the RL deconvolution with large datasets, with limited computing resources is challenging, but enables on-the-fly deconvolution. It is often desirable to process images while running an experiment to help locate precise 3D positions of beads or cells in light microscopy data, which is required before proceeding to the next experimental step (e.g., milling or sectioning). GPUs help boost processing speeds, but these are often more constrained in memory resources. RedLionfish by default, tries to use GPUs, but if an error occurs it will instead attempt to process using a *block iterative deconvolution* algorithm, whereby the data is split into smaller overlapping volumes and the full RL-deconvolution calculation is run independently for each chunk, and later merged into a single volume ^16^ ( Figure 2). Chunking data is a common technique when dealing with big datasets and is the main reason for the emergence of data processing software libraries such as *dask arrays* ^17^. Unfortunately, the nature of the RL deconvolution algorithm requires significant amounts of data from neighboring volumes which means that in order to get precise results after chunking, padding must be used and consequently edge-effects from each block will need to be taken into account (see below). It also helps reduce artifacts created by circularity introduced by the convolution based FFT. The padding can be programmatically adjusted in the block iterative algorithm implemented in RedLionfish with the parameter *psfpaddingfract* (set to 1.2 as default) in the function *block_RLDeconv3DReiknaOCL4*() within file *RLDeconv3DReiknaOCL.py*. This final padding value is equal to *PSF _paddingfract_* × *PSF _size_*, so each of the block edges will be cropped and merged to the final result data volume, essentially keeping only the central region, with the exception being at the edges of the full data block where there is no padding applied. See Figure 2, illustrating how the padding is used for merging calculation blocks.

There is no established criterion for the choice of number of iterations to use. This is usually adjusted by the user depending on either the visual quality of measurements, amount of noise in void areas or spatial precision of particular features. It should also be taken into consideration that a higher number of iterations leads to proportionally longer calculation time, and that too many iterations may result in the excessive spreading of artifacts (see section below).

### Operation

In order to run Redlionfish, there are no specific minimum requirements, but it is recommended that the computer have a CPU or GPU chip that supports OpenCL. If not, the algorithm has a fallback mechanism that uses of CPU only. It is also recommended that the memory available is large enough to be able to cope with the data being processed.

As explained in the previous section, there are different ways that Redlionfish can run Richardson-Lucy deconvolution on a volumetric dataset. With the napari interface it is as simple as selecting the data source and the PSF, and click on the “Go” button ( Figure 1). The calculation can take a while, and the result is then returned to the napari visualizer. This is the standard workflow of many napari plugins. For running programmatically there are many examples available in the GitHub repository *scripts* folder ( https://github.com/rosalindfranklininstitute/RedLionfish/tree/main/scripts), that demonstrate how to setup and run the deconvolution.

### Quality control

Unit tests can be run from the code root folder, with the command *pytest*. The *scripts* folder also contains a number of routines that can be used for testing or demonstrating how to use the package programmatically.

It is known in RL-deconvolution that edge errors propagate inwards due to the convolution operations in bounded volumes. Considering the one-dimensional case using data with *w _data_* contiguous number of points, a PSF with *w _PSF_* data points, and remembering that there are two discrete convolutions in each iteration of the RL algorithm, then the 'valid' region of the iteration result reduces by 2 *w _PSF_* – 2 per iteration. The 'valid' region is the data range where the iteration (double convolution) calculation does not rely on padded values at the boundaries, and it uses data points from the image and PSF only. This reduction in size is quite significant, for example, imagine that we can split our data into blocks with width of 512 and our PSF is 32. Then after a typical 10 iterations of the RL deconvolution, the valid region would be reduced to 512 – 10 × 2(2 × 32 – 2) = –108, which is less than the original data size, meaning that the whole result would be invalid. In this situation, the exact solution in the RL-deconvolution is only possible by running a very low number of iterations, which is often insufficient to restore the data to a useful level.

In fact, in most cases where users implement RL-deconvolution to restore data, several iterations are applied, well beyond the limit established for getting positive-sized valid regions. This is mainly due to the PSF having nearly zero or exponentially decaying values near the boundaries, which means that successive convolutions lead to decreasing dependency on near-edge data. RL deconvolution can work well in many cases where PSFs have the same size as the image data (see for example the *Caenorhabditis elegans* dataset example in Ref 18): the full result is collected without cropping to the 'valid'-only region. Theoretically the whole result is invalid but, in practice, despite the reduced precision in using this method, the results obtained are often accepted and considerably clearer of PSF optical artifacts. There are ways that the loss of quality can be mitigated, such as using edge normalization and block-interlacing methods ^16^, that work well in many cases such as when gaussian PSFs are used and with greyscale digital camera photos, however these are not mathematically correct. Non-circulant forms of convolutions often require data to be padded however this can introduce significant artifacts around the edges that propagate with the number of iterations if the padding is simply done with zeros or with the presence of noise that is not representative of the instrument noise. The circularization coming from FFT implementation of convolution avoids this problem as it automatically reuses its own data.

**Figure 3.**
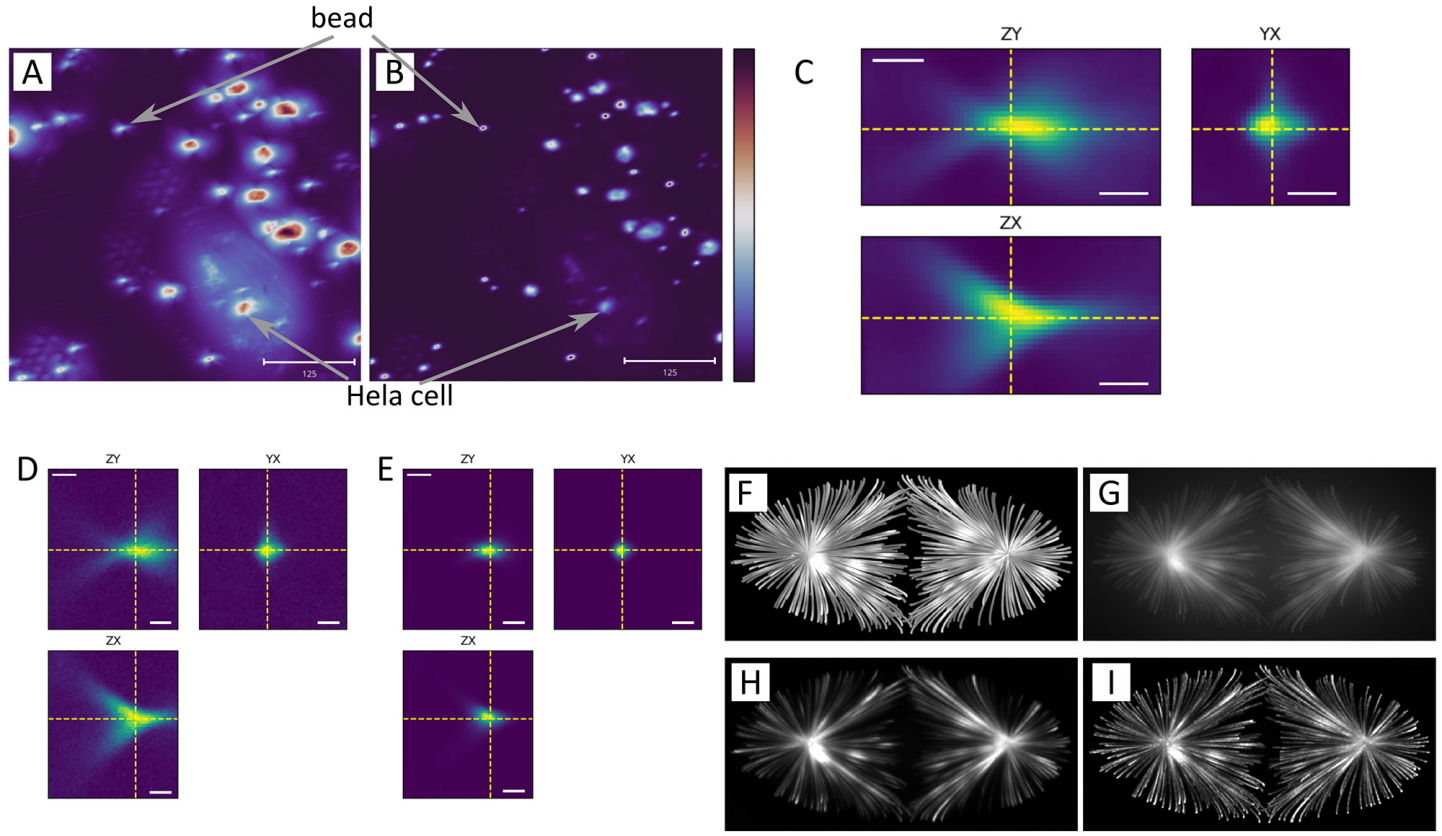
( **A**) Fluorescence volumetric data of cryo-preserved Hela cells transfected to express a fluorescent centriole marker (human CEP120-EGFP; Addgene plasmid #50382) and fluorescent beads imaged on a TEM grid. ( **B**) Resultant volume after running the RedLionfish deconvolution for 10 iterations, using the PSF shown in ( **C**). ( **A**) and ( **B**) scale bars are 125 voxels. ( **C**) This PSF data was averaged from several beads of the same volume dataset used in ( **A**) and it appears like an hourglass due to instrumental optical astigmatism. Ideally beads would appear point-like. ( **D**) Experimental bead data cropped from one of the beads in **A**, and ( **E**) after applying RedLionfish for 10 iterations using the PSF shown in **C**. After applying the RedLionfish deconvolution filter, noticeably the beads appear more spherical and cellular features appear to be clearer. ( **C**- **E**) Scale bars are 10 voxels. ( **F**- **I**) Publicly available dataset (512x256x128) of simulated Microtubules ^19^ and RedLionfish deconvolution results. ( **F**) Simulated data, set as ground truth. ( **G**) Convoluted data with a given PSF and added Poisson noise. ( **H**) RedLionfish after 100 iterations. ( **I**) RedLionfish after 500 iterations.

For examples that conduct deeper analysis of the effect that the number of iterations and PSF size have in reducing the valid region based on a precision criterion, see the RedLionfish notebooks contained in the repository and sublocation *scripts/block_vs_nonblock_maxwidth*. In the blocked processing algorithm, however each block will inevitably display these edge effects, which may or may not be visible, and so the padding fraction may need to be raised to provide a better result. Several solutions to mitigate this rapid reduction of the valid region were carefully considered but none of them was found to be feasible as they require significantly higher computer processing and memory consumption. This precision issue will be addressed in future versions of RedLionfish at the expense of speed in cases where it is required by the user. However, the current implementation is acceptable given that the resulting images are more detailed or provide better precision of beads positions in experimental data. See examples in Figure 3.

One interesting mathematical property of the Richardson-Lucy deconvolution algorithm is that, if both the data and PSF can be described by a gaussian function (with different amplitude, mean and width parameters), then a single iteration of the RL deconvolution results in data that is also described by a gaussian. For the one-dimensional case, the mathematics is demonstrated in the notebook *scripts/gauss_progress.ipynb*, and summarized here. If we assume that the original image data is point-like and experimentally convoluted with PSF, then the measured image is the same function as the PSF. In this simple case we can assume that the measured image and PSF is a one-dimensional gaussian with variance *r* ^2^, centered at zero and (to simplify) amplitude 1.


$$\;h(x)=e^{-\frac{x^{2}}{b^{2}}}\;(Eq. 6)$$

If we now assume that at a given RL iteration, the estimated image is also a gaussian with amplitude *R*, variance *b* ^2^ and centered at zero:


$$\;R_{n}=R.e^{-\frac{x^{2}}{r^{2}}}\;(Eq. 7)$$

Then, the next iteration of the RL algorithm, using Equation 5 results in:


$$\;\frac{\sqrt{\frac{b^{2}+r^{2}}{b^{2}r^{2}}}e^{\left(-\frac{r^{2}x^{2}}{b^{4}+2b^{2}r^{2}}-\frac{x^{2}}{r^{2}}\right)}}{\sqrt{\frac{b^{2}+2r^{2}}{b^{4}+b^{2}r^{2}}}}\;(Eq. 8)$$

which is also a gaussian with the variance given below, and interestingly the amplitude is independent of the amplitude of the previous iteration *R*:


$$\;\frac{1}{\frac{r^{2}}{b^{4}+2b^{2}r^{2}}+\frac{1}{r^{2}}}\;(Eq. 9)$$

Just to give an idea of how the RL algorithm progresses in 1D-gaussian-like data, with the added assumption that the measured image and PSF have variance equal to 1, then successive applications of the RL algorithm to gaussian-like estimated images, results in progressively decreasing relative gaussian variances of 75%, 49%, 23%, 5%, etc.

Several tests utilizing 3D gaussian data are elaborated in *scripts/RL_benchm_gauss_progress.ipynb*, which also compares Redlionfish with scikit’s, showing better performance on different metrics. Also, this interesting deconvolution-gaussian-mathematics makes it very useful to use it as a pytest runner, as is used in *test/test_consistency.py*.

The package is planned to be continuously supported as authors use it regularly to process their experimental data. The authors will maintain the repository, reply to any Github issues that arise and welcome contributions.

### Community acceptance

This package was well received by the scientific community, upon publishing it as a napari plugin in PyPI. It was shared by other scientists on Twitter and discussed in the image.sc forum ^20, 21^. There are other freely available RL-deconvolution software solutions available, and users are encouraged to try and compare ^22– 25^. RedLionfish combines many positive aspects by being user-friendly through napari, being reasonably fast, free and easily available, open source, and agnostic to PC hardware or operating system.

### Use cases

Figure 3 F-I shows an example of using Redlionfish for deconvolving simulated microtubules available in Ref 19, that has been artificially convoluted with a given PSF and added Poisson noise (G), then deconvoluted with 100 iterations (H) and 500 iterations with the same PSF (I). This is to illustrate how 3D data that is highly noised and convoluted by optical effects can be restored to a reasonable quality using the Richardson-Lucy deconvolution algorithm implemented in Redlionfish.

The Redlionfish GitHub package includes some scripts that can be used to benchmark the speed of the algorithm in its different forms (CPU and GPU) and also compares with scikit image restoration package. Table 1 was produced when running *scripts/test_and_benchm.py* on a laptop equipped with:

- 11th Gen Intel(R) Core(TM) i5-11300H @ 3.10GHz, 3110 MHz, 4 Core(s), 8 Logical Processor(s)
- 8Gb RAM
- Windows 11
- NVIDIA GeForce GTX 1650 with Max-Q Design (no dedicated VRAM), alongside Intel(R) Iris(R) Xe Graphics

**Table 1.** Benchmark result after running *test_and_benchm.py* script, with the missing detail in the blocking column completed manually. Blue shade highlights the result from a non-Redlionfish code for doing Richardson-Lucy deconvolution, that uses the scikit image implementation.

shape	method	GPU/CPU	blocking	iterations	time / s
(256, 256, 256)	doRLDeconvolutionFromNpArrays	CPU	No	10	5.6
(256, 256, 256)	doRLDeconvolutionFromNpArrays - OpenCL CUDA	GPU	No	10	3.9
(256, 256, 256)	nonBlock_RLDeconvolutionReiknaOCL - OpenCL CPU	CPU	No	10	3.8
(256, 256, 256)	skimage.restoration.richardson_lucy()	CPU	No	10	10.2
(1024, 1024, 64)	doRLDeconvolutionFromNpArrays - CPU	CPU	No	10	31.8
(1024, 1024, 64)	doRLDeconvolutionFromNpArrays - OpenCL CUDA	GPU	Yes	10	32.4
(1024, 1024, 64)	block_RLDeconv3DReiknaOCL - OpenCL CPU	CPU	Yes	10	22.0
(1024, 1024, 64)	skimage.restoration.richardson_lucy()	CPU	No	10	57.4

Please note that significantly different time results can be obtained in different systems. Typically, systems equipped with a GPU with dedicated VRAM will perform OpenCL calculations faster than the CPU OpenCL implementation. If users wish to study and exert more control of which algorithm to use, this can be done programmatically, and the *test_and_benchm.py* script can be very useful. All non-block GPU and CPU implementations give the same results, with the exception of when block implementation is used, which can give results with small differences.

This deconvolution algorithm was easily integrated in an existing 3D correlation software, to make it easier for users to quickly pre-process their data before running correlation optimizations. See for example https://github.com/rosalindfranklininstitute/3DCT.

## Ethics and consent

Ethical approval and consent were not required.

## Data Availability

RedLionfish runs in any system with python, and GPU. Functional OpenCL drivers are highly recommended as it can boost the speed of the deconvolution algorithm, otherwise it will run using the CPU backend. It has been tested successfully in Windows, macOS and Linux. It was written in Python programming language, minimum version recommended is 3.7. It has been tested successfully in an environment with the following packages: numpy, 1.24; scipy, 1.10; pyopencl, 2022.3; reikna, 0.8.0; Redlionfish is published in GitHub, https://github.com/rosalindfranklininstitute/RedLionfish, conda-forge and pypi. By being a registered napari plugin, it also means that it can be installed via the napari plugin installation interface. This package can be used programmatically and some usage examples are available in the GitHub repository, in jupyter notebook format or as python scripts. Source code available from: https://github.com/rosalindfranklininstitute/RedLionfish Archived software available from: https://doi.org/10.5281/zenodo.10854513 ^26^ License: Apache v2.0
