# Supplementary material for: RedLionfish – fast Richardson-Lucy Deconvolution package for efficient point spread function suppression in volumetric data
Source: Wellcome Open Res. 2024 Jun 3;9:296. [Version 1] doi: 10.12688/wellcomeopenres.21505.1 (PMC11413554; doi:10.12688/wellcomeopenres.21505.1)
Supplement: Peer review history

# Reviewer report: Reviewer response for version 1

**DOI:** 10.21956/wellcomeopenres.23776.r93961

**Published:** 2024-09-19

**Authors:** Axel Donath

**Affiliations:** 1 Center for Astrophysics |Harvard & Smithonian, Cambridge, USA

The authors present a new Python package named “RedLionfish”, which provides a high performance implementation of the well known Richardson-Lucy (RL) Deconvolution algorithm for volumetric data, as obtained by methods such as Fluorescence Light Microscopy. The paper is clearly written and targets a more general audience. The mathematical description stays on a superficial level, which is appropriate for a well known method such as the RL deconvolution and more general target audience. The package is openly developed on GitHub with unit tests and illustrative examples in form of Jupyter notebooks.

**Comments on the paper:**

- Check spelling of proper names, such as “Gaussian” or “Python”, they are often written lower case.
- Replace “convoluted” with “convolved”. The latter is the correct verb for describing the mathematical operation
- Around Eq(3) one should probably mention the Wiener Filter for completeness
- Just below Eq (5): the asterisk representing the convolution is not consistent with the one used in the equation above.
- Just below Eq (5): I think using “FSP” to denote a flipped PSF is somewhat un-unusual. Maybe an asterisk PSF* serves the purpose better.
- In Eq (6) and Eq (7) r^2 and b^2 are swapped and not consistent with the quantity mentioned in the descriptive text.

**Comments on the repository:**

- Consider documenting their public API. The code contains docstrings, but there is no public rendering available on a website.
- Consider providing instructions for contributions such as a CONTRBUTING.md file

Are the conclusions about the tool and its performance adequately supported by the findings presented in the article?

Yes

Is the rationale for developing the new software tool clearly explained?

Yes

Is the description of the software tool technically sound?

Yes

Are sufficient details of the code, methods and analysis (if applicable) provided to allow replication of the software development and its use by others?

Yes

Is sufficient information provided to allow interpretation of the expected output datasets and any results generated using the tool?

Yes

Reviewer Expertise:

Astrophysics with focus on low counts imaging methods.

I confirm that I have read this submission and believe that I have an appropriate level of expertise to confirm that it is of an acceptable scientific standard.

# Reviewer report: Reviewer response for version 1

**DOI:** 10.21956/wellcomeopenres.23776.r93964

**Published:** 2024-09-04

**Authors:** Haase Robert

**Affiliations:** 1 Data Science Center ScaDS, Uni Leipzig, Saxony, Germany

The manuscript I am reviewing presents RedLionFish, a Python library and napari plugin for GPU-accelerated image deconvolution. Image deconvolution is a crucial step in common image processing workflows. It enables removing imaging artifacts induced through the nature of microscopes. If used correctly, deconvolution can enhance image quality substantially, making the invisible visible. GPU-accelerating this process makes a lot of sense and I am sure the community appreciates the broad availability of the algorithms implemented in RedLionFish. The software is open-source, permissively licensed and all in all an excellent example of state-of-the-art good scientific practice in research software engineering. The paper is written in excellent language and scientifically sound. When reading it, I took some minor notes printed below. All of them can be considered as optional. It's just what came to my mind while reading the document. My suggestions may, or may not, improve the manuscript.

 Fantastic work!

 Sincerely,

 Robert Haase

 robert.haase@uni-leipzig.de

**Minor comments:**

**Page 3**, just below equation (3): Noise is certainly one cause of the described effect. Another practical issue in microscopy is that the PSF is often not exactly known. Many parameters we need to estimate, e.g. the refractive index of the imaged medium or of the sample itself.

**Page 3**, bottom right: Before using the abbreviation "GPU", consider writing it out once.

**Page 4**: "it is also cross-compatible with most CPUs and GPUs, and not restricted to NVIDIA cards" instead of mentioning one omnipresent hardware vendor, who develops their own standard (CUDA), consider mentioning some of the other big players behind OpenCL. The reader might appreciate explicit mentioning that your software is compatible with Apple / Intel / AMD hardware.

**Page 4**: "It is important to note here that OpenCL can run in both GPU and in CPU", in my experience support for CPUs is quite limited on the computer hardware market. As far as I know, only Apple M1/2/3 and Intel XEON support OpenCL to some degree. There is no broad support from hardware vendors anymore (unfortunately).

**Page 6**: " It is also recommended that the memory available is large enough to be able to cope with the data being processed." I presume you could be more precise. Assume my image is 1 GB large (uint16), how large should the GPU memory be to make RL work work (i.e. with out tiling)?

**Page 6**: "Quality control": Can you guide users who are new to deconvolution how to make sure that the deconvolution algorithm was applied correctly? Are there common artifacts one should look out for?

**Page 6**: "edge errors propagate inwards" - would it be possible to visualize this? This seems in particular relevant when processing the image in tiles. 

**Page 8**: " All non-block GPU and CPU implementations give the same results, with the exception of when block implementation is used, which can give results with small differences." I think this sentense could be simplified a bit, it mentiones the blocks twice with the same meaning/context.

Are the conclusions about the tool and its performance adequately supported by the findings presented in the article?

Yes

Is the rationale for developing the new software tool clearly explained?

Yes

Is the description of the software tool technically sound?

Yes

Are sufficient details of the code, methods and analysis (if applicable) provided to allow replication of the software development and its use by others?

Yes

Is sufficient information provided to allow interpretation of the expected output datasets and any results generated using the tool?

Yes

Reviewer Expertise:

Bio-image analysis, artificial intelligence, quantitative biology, data science

I confirm that I have read this submission and believe that I have an appropriate level of expertise to confirm that it is of an acceptable scientific standard.
